# The association between molar incisor hypomineralization and oral health-related quality of life: a cross-sectional study

**DOI:** 10.1007/s00784-022-04375-3

**Published:** 2022-02-11

**Authors:** K. Elhennawy, O. Rajjoub, D. R. Reissmann, M.-S. Doueiri, R. Hamad, I. Sierwald, V. Wiedemann, K. Bekes, P.-G. Jost-Brinkmann

**Affiliations:** 1 Department of Orthodontics, Dentofacial Orthopedics and Pedodontics, Charite – Universitatsmedizin Berlin, corporate member of Freie Universitat Berlin, Humboldt-Universitat zu Berlin, and Berlin Institute of Health, Berlin, Germany; 2 Department of Prosthetic Dentistry, Center for Dental and Oral Medicine, University Medical Center Hamburg-Eppendorf, Hamburg, Germany; 3 Private practice “MUNDWERK”, Berlin, Germany; 4 Department of Paediatric Dentistry, University Clinic of Dentistry, Medical University of Vienna, Vienna, Austria

**Keywords:** Molar incisor hypomineralization, MIH, Quality of life, Oral health-related quality of life, Dental management, Hypersensitivity, COHIP

## Abstract

**Objectives:**

We aimed to assess the association between molar incisor hypomineralization (MIH) and the oral health-related quality of life (OHRQoL) in a group of 7- to 14-year-old children in Berlin, Germany.

**Materials and methods:**

The cross-sectional study consisted of a consecutive sample of 317 children, aged 7–14 years (49% girls, 51% boys; mean age, 8.71). Data were collected between June 2018 and December 2019. MIH was diagnosed using the criteria of the European Academy of Paediatric Dentistry. OHRQoL was assessed using the German 19-item version of the Child Oral Health Impact Profile (COHIP-G19). Differences in COHIP-19 summary scores between controls without MIH and MIH patients and with regards to MIH severity were tested for statistical significance using *t* test and analysis of variance (ANOVA), respectively.

**Results:**

Data were obtained for 217 untreated MIH patients and 100 controls. OHRQoL of MIH patients was significantly more impaired than of controls indicated by COHIP-19 mean scores (60.9 ± 10.7 vs. 67.9 ± 7.8; *p* < 0.001). Patients with severe MIH (59.6 ± 11.0) reported significantly worse OHRQoL than patients with mild MIH (63.6 ± 9.1; *p* = 0.013).

**Conclusions:**

MIH has a significant negative impact on the children’s OHRQoL. Patients with severe MIH experience a greater negative impact on OHRQoL than those diagnosed with mild MIH.

**Clinical significance:**

MIH is one of the major dental problems of our time; pediatric dentists should be aware of its impact on the OHRQoL of the patient.

## Introduction

Molar incisor hypomineralization (MIH) was described as demarcated, qualitative developmental enamel defects of one or more of the permanent molars [1]. The terminology of MIH was first introduced by Weerheijm in 2001 [1]. The clinical characteristics can vary, both, between different patients and on tooth level in the same patient; however, no gender-related distribution differences have been reported [2]. The prevalence of such defects differs between countries and ranges from 2.4 to 40.2% worldwide [3].

Dental management of MIH represents a challenge for pediatric dentists, due to the variation in clinical appearance and the broad spectrum of treatment modalities, which range from prevention or restorations to extraction and orthodontic management [4]. The European Academy of Paediatric Dentistry (EAPD) guidelines for the treatment of MIH-affected teeth exist but without a relation to the oral health-related quality of life [4]. It was reported that the dental management need of affected children is much higher than in non-affected ones [5]. In severe MIH cases, a post-eruptive enamel breakdown (PEB) in the affected permanent molars is to be expected, because of the sub-surface porous structure and the detrimental effect of masticatory forces in this area [2, 6]. The post-eruptive breakdown of enamel encourages caries progression leading to pulpal involvement. This causes different degrees of hypersensitivity that range from occasional mechanical or thermal hypersensitivity to persistent/spontaneous hypersensitivity [1, 4]. In addition to the hypersensitivity, an aesthetic burden is obvious, in case of permanent incisor involvement, all of which may negatively affect the child’s general health, quality of life and socio-psychological status [4, 7].

Oral health-related quality of life (OHRQoL) is a multidimensional construct describing the patient’s self-perceived impact of oral health problems [8, 9]. Increased attention has lately been paid to the assessment of OHRQoL as an important part of the overall perceived general health [10, 11]. The influence of some dental conditions such as dental caries [12], malocclusion [13, 14] and tooth erosion [15] on the disturbance of a child’s daily activities has been addressed in the literature [10, 16]. Studying OHRQoL has proven to play an important role in planning public health policies and assessing different dental management modalities [9, 17]. The last decade has witnessed a great amount of research regarding the prevalence, diagnosis, management and etiology of MIH; however, only a few studies addressed the association between this condition and children’s OHRQoL worldwide [18–24]. Dantas-Neta reported on the negative impact of MIH on the OHRQoL of Brazilian 11–14-year-old school children [21]. Another Brazilian study done by Portella et al. (2018) aimed to study the association between MIH and the OHRQoL in 8–10-year-old school children from Curitiba, Brazil [23]. Their results were in line with those of Dentas-Neta et al., as they also found that MIH negatively affected the OHRQoL [21]. Gutiérrez et al. (2019) studied the impact of MIH on the OHRQoL of Mexican 8–10-year-old school children; in their cross-sectional study, they concluded that moderate/severe MIH-affected children experienced a lower quality of life compared to healthy children [19]. The interest in patient-reported outcome research is increasing in the dental community, which is also the case regarding the OHRQoL of MIH patients. However, we found a gap of knowledge on this topic in Germany, and thus, we aimed to determine the association between molar incisor hypomineralization and OHRQoL and, furthermore, to compare the OHRQoL of MIH children to healthy controls in Berlin, Germany.

## Materials and methods

### Subjects, study design and setting

In this cross-sectional study, 317 patients from the Department of Orthodontics, Dentofacial Orthopedics and Pedodontics at Charité – Universitätsmedizin Berlin and private dental practice in Berlin, Germany, were consecutively recruited between June 2018 and December 2019.

Both referred and in-house patients, who met the inclusion criteria, were consecutively recruited after routine clinical examination. To be included in the MIH group, children had to be 7–14 years old with minimum one MIH-affected first permanent molar. Only dentally untreated MIH-affected permanent first molars were included in the study. It was mandatory that the clinical tooth crown showed more than 1/3 of its length and the MIH lesion was required to be bigger than 1 mm in size. Children who could not read or speak German and/or those who already were dentally treated were excluded. The control group included all patients in the same age group, who had a routine check-up appointment and did not have any acute dental problems. After inclusion in the study, children were assigned to one of three groups: control, mild MIH or severe MIH.

The sample size was calculated for the primary outcome of this study, OHRQoL. We anticipated an effect size of 0.5 [25] and a standard deviation of 8 COHIP points [9]. Accordingly, to detect such an effect with a power of 90% and a type 1 error of 0.05, at least 85 patients are required for each group. Considering a potential drop-out rate of 20% and three groups for comparisons, a minimum required sample size of about 300 patients (100 per group) was eventually estimated.

The study has been approved by the Institutional Review Board of the Charité – Universitätsmedizin Berlin (EA2/104/16) and registered at DRKS.de (DRKS00011882). Written informed consent was obtained from all participants and one or both parents/caregivers prior to their enrollment.

### Oral examination

A full oral examination was performed at the first visit. MIH was diagnosed according to the European Academy of Paediatric Dentistry (EAPD) criteria: (a) demarcated opacities, (b) enamel disintegration, (c) atypical restoration, (d) tooth sensitivity and (e) extracted teeth [26]. All permanent teeth were clinically examined with a dental mirror and a dental explorer under standard dental unit lightning. The severity of MIH was classified according to Lygidakis et al. (2010) [5] as follows: mild, when there were demarcated opacities without post-eruptive breakdown, or as severe, when post-eruptive breakdown had occurred. The participant was graded as having a mild or a severe MIH according to the most affected MIH tooth. Moreover, data on confounding factors such as the number of affected teeth, hypersensitivity and the involvement of the permanent incisors was collected. Hypersensitivity was measured by cold air stimulus. The air was delivered with a standard dental unit air syringe at maximum pressure for 1 s from a distance of 1 cm perpendicular to the occlusal surface of the affected tooth. The children were clinically examined from one of three calibrated examiners, obtaining a Cohen’s kappa coefficient for inter-examiner calibration of 0.83.

### Assessment of oral health-related quality of life

Eligible participants were asked to complete the German version of the Child Oral Health Impact Profile (COHIP-G19) alone (without help of their parents). However, in case of not understanding a question, the study nurse explained the question again. This is a self-reporting oral health-related quality of life questionnaire, which measures both positive and negative impacts of oral conditions in children on their overall lives and has been used extensively in children’s oral health research [9]. The COHIP-G19 consists of 19 items encompassing three domains, oral health, functional well-being and socio-emotional well-being, and has been validated for use in 7–17-year-olds [9]. Participants are asked to report on the frequency of any impact over the past 3 months on a 5-point Likert scale, which is scored from 0 to 4 (never, rarely, sometimes, fairly often, almost all the time) with negative items having their score reversed. In addition, this instrument also has one general question concerning the participant’s perception of their overall oral health. The total score can range from 0 to 76, with the higher scores reflecting better OHRQoL [9].

### Statistical analysis

As the first part of the analyses, participants’ sociodemographic and oral health (MIH patients only) characteristics were assessed using means and standard deviations (SD) for continuous measures and frequencies and proportions for categorical measures. To test for statistically significant differences of these measures between groups, Student’s *t* test was applied for continuous data (age, number of affected teeth and surfaces) and chi-square test for categorical data (gender, anterior teeth affected, hypersensitivity).

In the second part of the analyses, the mean COHIP summary and dimension scores were compared between controls and MIH patients and with respect to MIH severity using Student’s *t* test and analysis of variance (ANOVA), respectively. For each between-groups difference of COHIP summary and dimension scores, the mean and corresponding 95% confidence interval and standardized effect size (ES) were computed. According to Cohen, an effect size above 0.2 indicates a small effect, above 0.5 indicates a medium effect, and above 0.8 indicates a large effect [27]. We considered an effect size of 0.5 as the threshold for clinical relevance as has been previously defined for self-reported health measures [25].

As third part of the analyses, the potential impact of MIH and MIH severity on OHRQoL (COHIP summary scores) was assessed with linear regression models statistically controlled for potential confounders (age, gender, severity, number of affected teeth). However, oral health characteristics were only included in the subgroup analysis of MIH patients.

All 19 COHIP items were complete in all controls (100.0%) and in 200 MIH patients (91.3%). Only 74 missing answers were observed in 19 MIH patients. In case of less than 50% missing information (up to nine items), scores for these items were replaced by the median of the remaining items within a participant containing sufficient information. All participants with 10 or more missing answers were excluded from the study. This resulted in a final sample size of 317 participants (100 controls and 217 MIH patients) available for analysis.

All statistical analyses were performed using the statistical software package STATA/MP (Stata Statistical Software, Release 14.2. StataCorp LP, College Station, TX, USA), with the probability threshold of a type 1 error set at 0.05.

## Results

### Characteristics of participants

Participants’ mean age was 8.7 ± 1.8 years. Controls were significantly older than MIH patients (*p* < 0.001) (Table 1). The percentage of girls and boys examined was 49% and 51%, respectively. The proportion of female participants was slightly higher in the controls than in the MIH group; this difference was, however, not statistically significant (*p* = 0.613).

**Table 1:** Characteristics of study participants

Variables	MIH			Controls *N* = 100
	Mild *N* = 60	Severe *N* = 157	All MIH *N* = 217	
	Mean (SD) or *N* (%)			
Age	8.2 (1.9)	8.3 (1.5)	8.3 (1.6)	9.7 (1.9)
Gender [female]	30 (49.2)	75 (47.5)	105 (48.0)	51 (51.0)
Affected incisors	41 (67.2)	132 (83.5)	173 (79.0)	-
Affected teeth	4.9 (2.2)	6.1 (2.4)	5.8 (2.4)	-
Hypersensitivity	14 (23.0)	110 (69.6)	124 (56.6)	-

More than two-thirds (72%) of the MIH group participants had a severe form of MIH according to EAPD criteria. Six teeth (3 anterior teeth) and 11 surfaces were affected on average. Moreover, patients with severe MIH-affected teeth showed more involved MIH teeth in total (*p* = 0.001) than those with mild ones, more anterior teeth (*p* = 0.014) and more surfaces (*p* < 0.001). The anterior teeth were affected in 79.0% of MIH cases, with cases of severe MIH being affected significantly more frequently (*p* = 0.008). Hypersensitivity was reported in just over half (56.6%) of MIH cases, with significantly more cases of severe MIH (*p* < 0.001). The characteristics of these 317 participants are summarized in Table 1.

### OHRQoL and MIH

On average, overall OHRQoL was significantly more impaired in MIH patients than in controls indicated by a substantial difference in mean COHIP summary scores (MIH patients, 60.7 points; controls, 67.9 points; *p* < 0.001) (Table 2). A lower OHRQoL in MIH patients was also observed for all three COHIP subscales with the greatest difference for the *oral health* subscale, followed by the *functional well-being* subscale and the *social/emotional, school and self-image subscale* (all *p* < 0.001). All differences in summary and subscale scores can be rated as clinically relevant based on the ES ranging from 0.46 to 0.97.

**Table 2:** COHIP-19 subscales and summary scores for all participants and stratified for MIH

COHIP subscales	All	Controls	MIH			
	*N* = 317	*N* = 100	*N* = 217			
	*Mean (SD)*	*Mean (SD)*	*Mean (SD)*	*Diff (95% CI)*	*ES*	*p value*
COHIP-G19 oral health subscale	13.9 (3.6)	16.0 (3.0)	12.8 (3.4)	3.2 (2.4; 4.0)	0.97	< 0.001
COHIP-G19 functional well-being subscale	14.0 (2.6)	14.9 (2.1)	13.6 (2.7)	1.3 (0.7; 1.9)	0.52	< 0.001
COHIP-G19 social/emotional, school and self-image subscale	35.1 (6.2)	37.0 (4.3)	34.3 (6.7)	2.8 (1.3; 4.2)	0.46	< 0.001
COHIP-G19 summary score	63.0 (10.4)	67.9 (7.8)	60.7 (10.7)	7.3 (4.9; 9.6)	0.74	< 0.001

When only MIH patients were considered, patients with severe MIH had a significantly worse OHRQoL than those with a mild one indicated by the substantial difference in mean COHIP summary scores (severe MIH, 59.6 points; mild MIH, 63.6 points; *p* = 0.013) (Table 3). However, according to the ES ranging from 0.27 to 0.38, the effect was only moderate and likely not clinically relevant.

**Table 3:** COHIP-19 subscales and summary scores according to MIH severity

COHIP subscales	MIH mild	MIH severe			
	*N* = 60	*N* = 157			
	Mean (SD)	Mean (SD)	*Diff (95% CI)*	*ES*	*p value*
COHIP-G19 oral health subscale	13.5 (3.3)	12.6 (3.5)	0.9 (− 0.1; 1.9)	0.26	0.082
COHIP-G19 functional well-being subscale	14.1 (2.5)	13.4 (2.7)	0.7 (− 0.1; 1.5)	0.27	0.081
COHIP-G19 social/emotional, school and self-image subscale	36.0 (5.0)	33.6 (7.1)	2.4 (0.4; 4.3)	0.36	0.019
COHIP-G19 summary score	63.6 (9.1)	59.6 (11.0)	4.0 (0.8; 7.1)	0.38	0.013

Differences in COHIP summary scores between controls and MIH patients and with respect to MIH severity were also confirmed when all three groups (controls, mild MIH, severe MIH) were collectively analyzed in an ANOVA (*p* < 0.001).

When the impact of MIH severity was assessed in linear regression models, patients with mild MIH had on average 4.4 points lower COHIP summary scores than controls, while the impact of severe MIH on OHRQoL was almost twice as much (Table 4, Model #1). These values changed only slightly and not significantly when statistically controlled for participants’ age and gender (Table 4; Model #2).

**Table 4:** Impact of MIH on OHRQoL in linear regression analysis (Model #1) and statistically controlled for potential confounders (Model #2)

Predictor	Coefficient	95% CI	*p* value
*Model #1*			
Control^*^	-	-	-
Mild MIH	− 4.4	− 7.5; − 1.2	0.006
Severe MIH	− 8.4	− 10.8; − 5.9	< 0.001
*Model #2*			
Control^*^	-	-	-
Mild MIH	− 4.6	− 7.9; − 1.3	0.006
Severe MIH	− 8.6	− 11.2; − 6.0	< 0.001
Age [y]	− 0.1	− 0.8; 0.5	0.650
Gender [female]	− 0.5	− 2.7; 1.7	0.653

* Reference category

Among MIH patients, the diagnosis of severe MIH was associated with on average 4.0 lower COHIP summary scores compared to patients with mild MIH (Table 5; Model #3). Values stayed virtually identical when statistically controlled for participants’ age and gender (Table 5; Model #4). However, when additionally controlled for oral health characteristics (i.e. number of affected teeth and presence of hypersensitivity), the impact of the diagnosis of severe MIH compared to mild MIH decreased to only 1.6 COHIP points (Table 5; Model #5). The impact of MIH severity was substantially moderated by the number of affected teeth. That is, the COHIP summary score was on average one point lower for each affected tooth.

**Table 5:** Impact of MIH severity on OHRQoL in linear regression analysis (Model #3) and statistically controlled for age and gender (Model #4) and additionally for dental health (Model #5)

Predictor	Coefficient	95% CI	*p* value
*Model #3*			
MIH severity	− 4.0	− 7.1; -0.8	0.013
*Model #4*			
MIH severity	− 4.0	− 7.1; -0.8	0.014
Age [y]	− 0.4	− 1.2; 0.5	0.422
Gender [female]	− 0.9	− 3.7; 2.0	0.548
*Model #5*			
MIH severity	− 1.6	− 5.0; 1.8	0.344
Age [y]	− 0.2	− 1.0; 0.7	0.683
Gender [female]	− 0.3	− 3.1; 2.5	0.827
Number of affected teeth	− 1.0	− 1.6; -0.4	0.001
Presence of hypersensitivity	− 2.6	− 5.7; 0.5	0.104

## Discussion

To the best of our knowledge, this is the first study evaluating the association between MIH and OHRQoL in Germany. Several authors studied the effect of MIH on OHRQoL in different countries in the world [18–24], most of which used the Child Perceptions Questionnaire (CPQ). In contrast to other studies, we used the validated German short version of the COHIP questionnaire (COHIP-G19) [9]; one of the reasons for that was the lack of another validated German version questionnaire existing at the time of planning the study. The original COHIP is a 34-item questionnaire that has been established to measure the OHRQoL in children and adolescents at the age of 8 to 15 years [17]. The questionnaire is divided into 5 subscales (oral health, school environment, self-image, emotional well-being and functional well-being). It is the first OHRQoL measurement tool for children to include both the positive and negative health impacts. Recently a 19-item short version of COHIP has been introduced by Broder et al. [17], which was then translated and validated by Sierwald et al. [9], giving rise to the German 19-item short version (COHIP-G19) [9]. The short version of the COHIP is advantageous in many ways; it is faster and easier to be administered, which is more convenient for the young respondents. Moreover, it covers a wider age range (7–17 years) in comparison to the age range validated using the long form (8–15 years) [9, 17]. For our sample, we found the age range 7–14 years old appropriate, since it falls in the validated age range of the validated COHIP-G19 questionnaire and children over the age of 14 are very unlikely to still have untreated affected teeth. Moreover, this age range goes in line with most published studies reporting on the association of MIH and OHRQoL [18–24].

There is only one study that also used this questionnaire. Hasmun et al. (2018) reported on the positive change of quality of life of MIH patients after the treatment of MIH incisor lesions [20]. The study included 111, 7–16-year-old children. Before treatment, the children showed a lower OHRQoL compared to our sample. This might be explained with the inclusion criteria of the study. Hasmun et al. included only children, who were besides being diagnosed with MIH and showing a visible enamel opacity involving at least one permanent incisor, also requesting improvement in their incisor aesthetics. In contrast, we included all patients suffering from MIH in different severities.

Further studies evaluating the impact of MIH on OHRQoL and using the CPQ as an instrument show that children suffering from this disease show impaired OHRQoL. For example, the impact of MIH on OHRQoL was previously evaluated in a sample of 88, 7–10-year-old Columbian students using the CPQ8–10, who reported a negative impact of MIH on the OHRQoL in all four domains [22]. Interestingly, the severity of MIH did not differ statistically between groups with relation to the domains of the questionnaire and overall score, possibly because very few participants presented with severe MIH. Furthermore, this study had some drawbacks like not taking several confounding factors into their analysis. A study done in Mexico on a sample of 411, 8–10-year-old schoolchildren also using the CPQ8–10 confirmed the negative impact on MIH-affected children’s OHRQoL [19]. Children with moderate/severe MIH experienced a greater impact across the four domains compared to children without MIH. A study by Portella et al. (2019) of 728, 8–year-old children also using the CPQ8–10 reported a significant negative impact of MIH on the oral symptom domain [23].

Another study carried out in Brazil of 594, 11–14-year-old children using CPQ11–14 reported a significant negative impact of MIH on the oral symptoms and functional limitation domains [21]. Despite using another OHRQoL questionnaire, our results are in line with those studies. We were also able to show that the OHRQoL was significantly more impaired in MIH patients than in controls indicated by substantial differences in mean COHIP total summary score as well as in all three domains. Most recently, Dias et al. 2020 found the same results with a group of 253 children aged 6–12 years in Brazil. This study used both self-reporting versions of the Child Perceptions Questionnaire, the 8–10 and the 11–14 versions, respectively, and the Parental-Caregiver Perceptions Questionnaire (P-CPQ) [18].

Some studies also reported that a higher prevalence of female children presented an impact on OHRQoL in all four or some domains when applying the CPQ compared with male children [18, 23]. The authors assumed that girls might have greater concerns with oral and aesthetic health problems as already shown in other studies [28]. However, our results did not show any differences between girls and boys.

### Strengths and limitations of the study

One of the limitations of our study might be the cross-sectional study design; this design does not measure the effect prospectively but at a certain point in time in the life of the participant. However, this study design is acceptable and widely used, especially in those types of studies, where a prospective design is practically very challenging. Furthermore, the study population was not representative for all children in Germany, since we only recruited patients living in Berlin and who visited one of the two recruitment dental centers. Another point is that the statistical control for hypersensitivity should be viewed critically since the corresponding symptoms are part of the quality of life. However, if the variable was not included in the model, the results did not change significantly. There was also a statistically significant age difference between the MIH group and the control group that might be considered as a limitation of our results. However, the impact of MIH on OHRQoL was statistically controlled for age with no significant change in the findings. Another possible limitation of the study might be the ability of children in the age range (especially the younger ones) to recall and/or answer questions about the impact over the last 3 months. However, the applied instrument is not only validated, but there is also sufficient evidence suggesting that children in this age group can accurately reflect their oral health impairments, sometimes even better than their proxies [29, 30]. Other confounding factors such as overall caries experience and severity of malocclusion could also influence the results; however, we tried to concentrate on MIH-affected molars and the direct confounding factors affecting MIH lesions, to specify our results as much as possible.

A strength of the study is that we applied a validated and internationally accepted instrument to assess OHRQoL in children and adolescents, the COHIP-G19. Another important aspect is that the study sample was recruited from a mixed university hospital/private practice setting, aiming for better external variability and generalizability. Furthermore, participants were recruited consecutively to limit selection bias and diagnosed by calibrated dentists.

### Future research recommendations

Future MIH research in this area needs to consider a longitudinal approach. The prospective dental treatment effect on these patients needs to be discussed as well.

## Conclusion

In conclusion, MIH has a substantial negative impact on children’s OHRQoL. Severe MIH is associated with a greater negative impact on OHRQoL than mild MIH. Moreover, more patient-centerd outcomes, such as OHRQoL, should be addressed in future research.
