# Correction to: The association between molar incisor hypomineralization and oral health‑related quality of life: a cross‑sectional study

**DOI:** 10.1007/s00784-022-04523-9

**Published:** 2022-04-30

**Authors:** K. Elhennawy, O. Rajjoub, D. R. Reissmann, M.-S. Doueiri, R. Hamad, I. Sierwald, V. Wiedemann, K. Bekes, P.-G. Jost-Brinkmann

**Affiliations:** 1 Department of Orthodontics, Dentofacial Orthopedics and Pedodontics, Charite - Universitatsmedizin Berlin, corporate member Freie Universitat Berlin, Humboldt-Universitat Zu Berlin, and Berlin Institute of Health, Berlin, Germany; 2 Department of Prosthetic Dentistry, Center for Dental and Oral Medicine, University Medical Center Hamburg-Eppendorf, Hamburg, Germany; 3 Private Practice “MUNDWERK”, Berlin, Germany; 4 Department of Paediatric Dentistry, University Clinic of Dentistry, Medical University of Vienna, Vienna, Austria

**Correction to: Clinical Oral Investigations**

https://doi.org/10.1007/s00784-022-04375-3

In the published paper, the affiliations of the authors are not correct.

The original article has been corrected.

